# Stable Ti_3_C_2_ MXene-Based Nanofiltration Membrane Prepared by Bridging for Efficient Dye Wastewater Treatment

**DOI:** 10.3390/membranes15110343

**Published:** 2025-11-18

**Authors:** Yu Zhang, Ming Qiu

**Affiliations:** College of Biological Chemical Science and Engineering, Jiaxing University, Jiaxing 314001, China

**Keywords:** Ti_3_C_2_ membrane, bridging, polyethyleneimine, polydopamine, stability, dye wastewater treatment

## Abstract

Transition metal carbides/nitrides (MXenes) nanosheets have emerged as promising candidates for constructing high-performance nanofiltration (NF) membranes for separation processes. However, MXene membranes exhibit limited feasibility due to the instability of their microstructure, which can lead to failure in the filtration process. This study presents a bridging strategy (polyethyleneimine and polydopamine) to prepare a stable titanium carbide (Ti_3_C_2_) membrane, resulting in superior nanofiltration efficiency. Polyethyleneimine intercalation can inhibit the tendency to swell, while polydopamine enhances the force between the substrate and nanosheets. The optimized membrane possesses a permeate flux of 112.3 L m^−2^ h^−1^ bar^−1^ (1.6 times higher than pristine Ti_3_C_2_ membrane) and good selectivity (methyl blue rejection rate: ~99.5%; Na_2_SO_4_ rejection rate: <5.0%). In addition, the prepared membrane has good long-time durability and is more suitable for low pressure nanofiltration. Notably, the bridging strategy is also applicable to various two-dimensional lamellar membranes. This strategy provides a universal method for enhancing the stability of two-dimensional membranes, thereby promoting their practical applications in robust separation processes.

## 1. Introduction

The textile sector produces significant amounts of wastewater rich in dyes and salts, which present considerable risks to both human health and society [1,2,3]. Membrane technologies are extensively employed in processes of separation and purification, given their benefits, including high efficiency in separation, eco-friendliness, and energy conservation [4,5]. Notably, nanofiltration (NF) technology has been demonstrated as effective for treating textile wastewater [3,6,7]. The core of NF technology is high-performance NF membrane material [8,9,10]. However, traditional thin-film composite polyamide membrane still has many problems, such as the permeability–selectivity trade-off, which limits its application in the precise separation of molecules and ions [11,12,13].

The utilization of two-dimensional (2D) materials for the fabrication of membranes has attracted considerable interest in recent studies [14,15]. Owing to the atomic-scale thickness of 2D nanosheets, ultrathin membranes with nanosized interlayer channels can be produced, enabling rapid and selective molecular transport [16,17]. The development of 2D nanomaterials used in the creation of NF membranes has mainly been with graphene oxide (GO), molybdenum disulfide (MoS_2_), transition metal carbides/nitrides (MXenes), metal–organic frameworks (MOFs), and covalent organic frameworks (COFs) [18,19,20,21,22]. As the most studied MXene phase, 2D titanium carbide (Ti_3_C_2_) possesses the merits of large lateral dimensions, ease of functionalization, good hydrophilicity, and antibacterial properties, and so has emerged as one of the most favored materials for constructing high-performance NF membranes [23,24,25]. However, the performance of Ti_3_C_2_-based membranes is often hindered, especially in water-related applications, due to the swelling effect of the nanosheets, which leads to the membrane’s susceptibility to disintegration in aqueous environments [26,27,28]. Therefore, it is essential to enhance the stability of Ti_3_C_2_-based membranes to effectively transfer their advantageous structural properties to water-related applications.

Many strategies have been proposed to improve the stability of Ti_3_C_2_ nanosheets, including crosslinking, cation intercalation, and self-crosslinking [26,29,30,31]. These methods significantly improve the interaction among the neighboring nanosheets. Lu et al. [28] prepared a self-crosslinked Ti_3_C_2_ membrane by establishing Ti−O−Ti bonds between the adjacent nanosheets through a simple thermal treatment process. This self-crosslinked Ti_3_C_2_ membrane demonstrates remarkable stability and effective ion exclusion. Wang et al. [32] prepared a Ti_3_C_2_ laminar membrane with nanochannel diameters of 7.4  Å by Ca-alginate hydrogel pillars. The resulting membrane presents an excellent sieving property towards valent cations (100% for Na_2_SO_4_). Zhang et al. [33] prepared a poly (acrylic acid)-modified Ti_3_C_2_/polyacrylonitrile composite membrane. The formed semi-interpenetrating network structure of poly (acrylic acid) can stabilize the structure and tune the d-spacing of the Ti_3_C_2_ membrane. Thus, this composite membrane shows superior permeability (516.34 L m^−2^ h^−1^) and high dye rejection (99.52%). Our previous work reported a tannic acid–iron-modified Ti_3_C_2_ membrane, which exhibits high water flux (90.5 L m^−2^ h^−1^ bar^−1^) and good separation efficiency for treating dye wastewater [23]. These modification methods not only inhibit the swelling of the nanosheets but also adjust the layer spacing of the membrane, thereby enabling the precise separation of ions or molecules. In addition, the laminar structure of nanosheets can be readily detached from the substrate during cross-flow testing conditions, significantly compromising the operational durability of 2D membranes [34,35,36]. To enhance the stability of the membrane, it is essential to simultaneously reinforce the interlaminar and interfacial interactions of the Ti_3_C_2_ membrane.

In this study, the hierarchical molecular bridging method was used to stabilize the Ti_3_C_2_ MXene membrane. Polyethyleneimine (PEI) was mixed with Ti_3_C_2_ solution, and then Ti_3_C_2_-based membranes were prepared on a polydopamine (PDA)-modified polyethersulfone substrate via vacuum filtration. The PEI polymer can act as a physical molecular bridge to restrain the swelling effect by hydrogen bonding and electrostatic force. Meanwhile, the d-spacing of the 2D membrane can be modified by adjusting the mass ratio of PEI to Ti_3_C_2_, which endows the membrane with a combination of high selectivity and permeability. In addition, the existence of PDA coating on the substrate can enhance the interactions between the Ti_3_C_2_ laminate and the substrate. Through the rational design of the molecular bridge, Ti_3_C_2_-based membranes have been stabilized and present excellent durability.

## 2. Experimental

### 2.1. Materials

Polyethersulfone (PES) membranes with a pore size of 0.22 μm, lithium fluoride (LiF, 99%), alcian blue 8GX (AB8GX, 50%), methyl blue (MB, AR), Congo red (CR, 98%), chrome black T (CBT, IND), methyl orange (MO, IND), methylene blue (MeB, 98%), and Ti_3_AlC_2_ powder (400 mesh, 99.5%) were sourced from Titanchem Co., Ltd., Shanghai, China. Hydrochloric acid (HCl, 37%), sodium sulfate (Na_2_SO_4_, 99%), sodium chloride (NaCl, 99.99%), magnesium sulfate (MgSO_4_, 98%), and magnesium chloride (MgCl_2_, 99%) were acquired from Sinopharm Chemical Reagent Co., Ltd., Shanghai, China. Additionally, polyethyleneimine (Mw = 10,000, 99%), dopamine hydrochloride (98%), and tris (hydroxymethyl) aminomethane hydrochloride buffer (Tris-HCl, 0.01 M, pH 8.5) were obtained from Macklin Reagent Co., Ltd., Shanghai, China. All chemicals were used as received, and deionized water was employed throughout the entirety of this study.

### 2.2. Preparation of Ti_3_C_2_

The synthesis of Ti_3_C_2_ nanosheets was achieved employing a mild etching method [37]. Initially, 1.0 g of lithium fluoride (LiF) was mixed with 10.0 mL of 9.0 mol/L hydrochloric acid (HCl) under magnetic stirring in a tetrafluoroethylene beaker at room temperature until complete dissolution occurred. Afterward, 0.5 g of Ti_3_AlC_2_ powder was incrementally introduced into the acidic solution. This mixture was stirred continuously at 35 °C for a duration of 24 h. Subsequently, the product underwent centrifugation at 3500 rpm for 5 min and was washed with deionized (DI) water until the pH rose above 6. The resulting product was then re-dispersed in water using ultrasonic treatment. Finally, the resultant suspension was centrifuged at 3000 rpm for 30 min; the supernatant was then collected and refrigerated for future use.

### 2.3. Preparation of Ti_3_C_2_ Membranes

A total of 0.4 g of dopamine hydrochloride was dissolved in 200.0 mL of Tris-HCl buffer solution under stirring at room temperature to create a uniform solution. The PES membranes were placed in the dopamine solution and allowed to incubate for 2 h, enabling the development of a polydopamine layer on the membrane surface.

**Figure 1:**
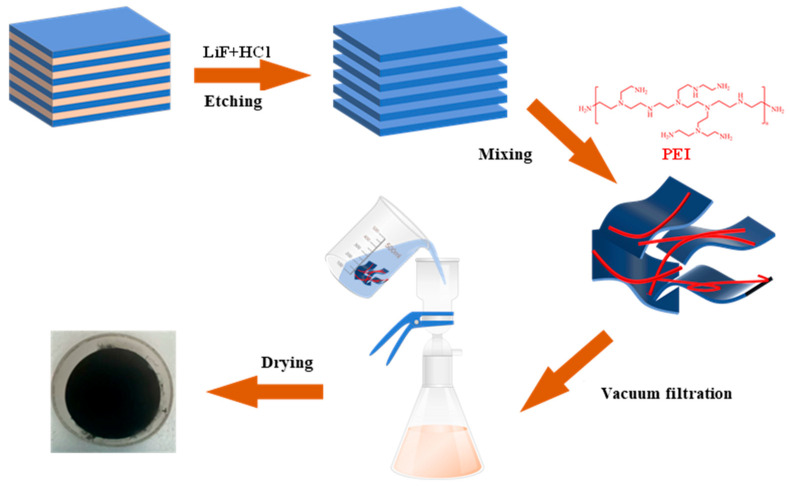
Schematic illustrations depicting the preparation procedure of the membrane.

Subsequently, polyethyleneimine (PEI) of varying mass was added to 50.0 mL of Ti_3_C_2_ solution, followed by ultrasonic treatment for 0.5 h and stirring for 1 h to obtain a uniform suspension. Membranes based on Ti_3_C_2_ and PEI were subsequently fabricated through a vacuum filtration approach and then dried in a vacuum oven (Figure 1). These membranes were labeled MPx, with M signifying Ti_3_C_2_ MXene, P indicating PEI, and X representing the mass ratio of PEI to Ti_3_C_2_. Table 1 provides the detailed composition of the produced membrane.

### 2.4. Instrument

The morphology of the Ti_3_C_2_ nanosheets was analyzed through transmission electron microscopy (TEM, FEI, TalosF200X, Waltham, MA, USA). The zeta potential of the nanosheets was determined with an electrokinetic analyzer (Anton Paar, SurPASS, Graz, Austria). For evaluating the structural properties of the Ti_3_C_2_ membranes, X-ray diffraction (XRD, Panalytical X’Pert3 Powder, Almelo, The Netherlands) was used, utilizing Cu Kα radiation (λ = 0.15418 nm) with a step size of 1.0°. The d-spacing value was determined by utilizing Bragg’s law: λ = 2dsinθ. The chemical functional groups in both the nanosheets and membranes were examined via Fourier transform infrared spectroscopy (FTIR, Thermo Fisher Scientific, IS50, Waltham, MA, USA) across a spectral range from 4000 to 500 cm^−1^. The chemical composition of the membranes was evaluated using X-ray photoelectron spectroscopy (XPS, Thermo Fisher Scientific, K-Alpha, Waltham, MA, USA). The surface and cross-sectional structures of the membranes were further assessed using scanning electron microscopy (SEM; S4800, Hitachi, Tokyo, Japan). Samples were placed on aluminum stubs and coated with gold before testing. To obtain topographical images of the membranes, atomic force microscopy (AFM; Bruker Dimension Icon, Karlsruhe, Germany) was employed in tapping mode. The quantitative roughness of the surface was evaluated using NanoScope Analysis V3.0 software, expressed as mean roughness (Ra). All samples were dried and stored in a dryer before testing. The experimental data were processed and visualized using Origin 2019 software.

### 2.5. Separation Performance of the Ti_3_C_2_ Membrane

The performance of membrane filtration was evaluated using a laboratory-scale cross-flow filtration system, which had a functional filtration area of 3.14 cm^2^. The test temperature is 25 °C.

The permeate flux (F, L m^−2^ h^−1^) for the membranes was derived from Equation (1).

F = V/A t(1)

In this equation, V (L) denotes the volume of the filtered water, t (h) represents the duration, and A (m^2^) signifies the effective filtration area.

The membrane rejection rate to dyes or salts was determined via Equation (2).

R (%) = 100(C_0_ − C_f_)/C_0_ (2)

Here, C_f_ (g L^−1^) and C_0_ (g L^−1^) refer to the concentrations of the filtered and feed solutions, respectively. The concentrations of dyes were evaluated by assessing the absorbance of the dye solution at its maximum absorption wavelength using a UV-vis spectrophotometer (UV 7, Mettler Toledo, Columbus, OH, USA). Meanwhile, salt concentrations were ascertained through conductivity measurements with a conductivity meter (FE 38, Mettler Toledo, Columbus, OH, USA).

The stability of the membrane was assessed by a long-term filtration measurement, with a duration of 72 h and an operational pressure set at 3.0 bar.

Each sample underwent testing a minimum of three times, and the average result was considered the final outcome.

## 3. Results

### 3.1. Characterization of Ti_3_C_2_

**Figure 2:**
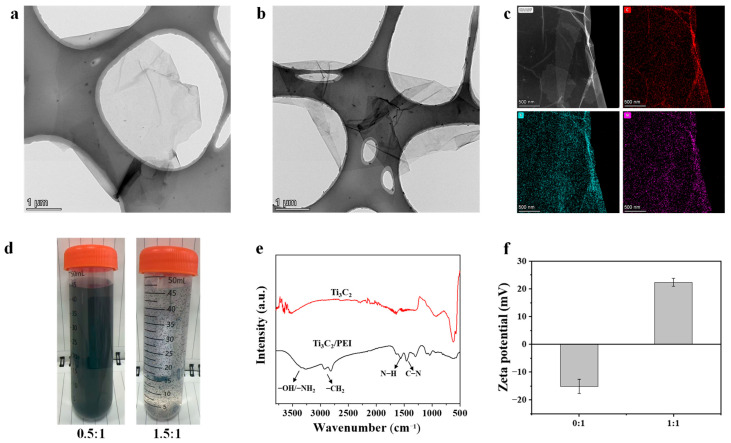
(**a**) TEM image illustrating Ti_3_C_2_ nanosheet; (**b**) TEM image illustrating PEI-modified Ti_3_C_2_; (**c**) STEM-EDX elemental mapping images of PEI-modified Ti_3_C_2_ (PEI/Ti_3_C_2_ = 1:1); (**d**) Photos of PEI/Ti_3_C_2_ mixture with different mass ratio; (**e**) FTIR spectra of Ti_3_C_2_ and PEI-modified Ti_3_C_2_ (PEI/Ti_3_C_2_ = 1:1); (**f**) Zeta potential of PEI/Ti_3_C_2_ mixture at pH = 6.5.

As shown in Figure 2a, single-layer Ti_3_C_2_ nanosheets measuring 3.0−4.0 μm in lateral dimensions can be acquired after the etching treatment. After modification with PEI, the nanosheets retain their morphological features without any considerable alteration (Figure 2b). The elemental composition of the nanosheets was further analyzed using dispersive X-ray spectroscopy (EDX). As shown in Figure 2c, the nitrogen element was uniformly distributed on the nanosheet surface. However, when the mass ratio of PEI/Ti_3_C_2_ reached 1.5:1, sediments appeared in the mixed solution due to electrostatic interactions between nanosheets and PEI (Figure 2d). As shown in Figure 2e, the FTIR spectrum of Ti_3_C_2_ displays absorption peaks at 3440 cm^−1^ and 1630 cm^−1^, which are associated with the stretching vibrations of hydroxyl (−OH) and carbonyl (C=O) functional groups, respectively. For the PEI-modified Ti_3_C_2_, three additional peaks at 2830 cm^−1^, 1580 cm^−1^, and 1362 cm^−1^ can be observed, which correspond to the characteristic vibrations of −CH_2_, N−H, and C−N bonds from PEI [38], respectively. Furthermore, compared to the unmodified Ti_3_C_2_, the −OH peak in the spectrum of PEI-modified Ti_3_C_2_ shifts to a lower wavenumber, suggesting that hydrogen bonding has occurred between the nanosheets and the PEI molecules [17]. The zeta potential of the nanosheet suspension was additionally assessed (Figure 2f). The Ti_3_C_2_ suspension consistently maintains a negative charge because of the presence of oxygen-containing functional groups on the nanosheet surface, while the PEI/Ti_3_C_2_ suspension is positively charged at pH = 6.5. Due to the high density of positively charged amino groups on polyethyleneimine (PEI) molecular chain, a portion of these groups neutralize the negative surface charge of the Ti_3_C_2_ nanosheets. The remaining amino groups are exposed to the surrounding medium, which causes the PEI/Ti_3_C_2_ nanosheets to exhibit a positive surface charge [39]. These results support the successful combination of Ti_3_C_2_ with PEI molecules.

### 3.2. Characterization of Membranes

**Figure 3:**
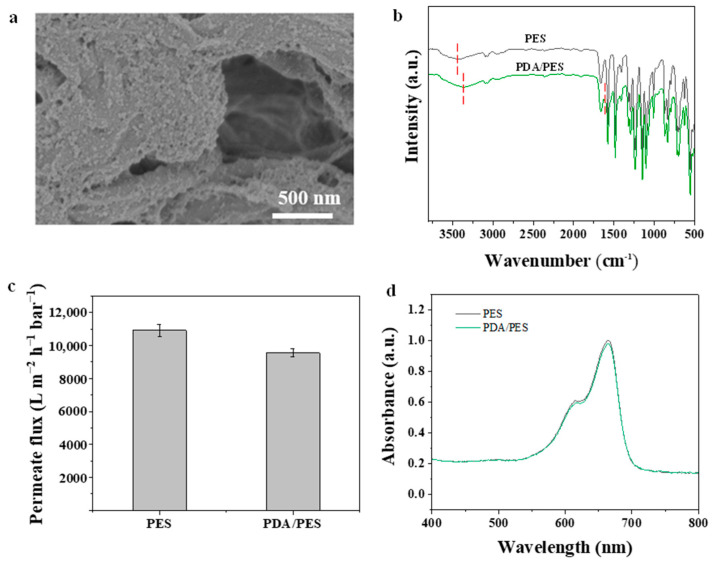
(**a**) Surface SEM image of PDA-modified PES membrane; (**b**) FTIR spectra of PES and PDA-modified PES membrane; (**c**) Permeate flux of PES and PDA-modified PES membrane; (**d**) UV−Vis absorption spectrum of methyl blue solution after filtration by PES and PDA-modified PES membrane.

As illustrated in Figure 3a, many granular protrusions can be observed on the PDA-modified membrane surface. The FTIR analysis of the surface chemical structure of the membranes is depicted in Figure 3b. Compared to the original PES substrate, the PDA-coated membrane displays a peak at 1580 cm^−1^, which is associated with the N-H bending vibration characteristic of polydopamine [40]. These findings imply a successful PDA modification. Moreover, the presence of PDA improves the interaction between the PES membrane and Ti_3_C_2_ laminate. In addition, after PDA modification, the flux of the membrane decreases slightly, and the dye rejection has almost no change, indicating that the PDA layer has minimal influence on the separation performance.

**Figure 4:**
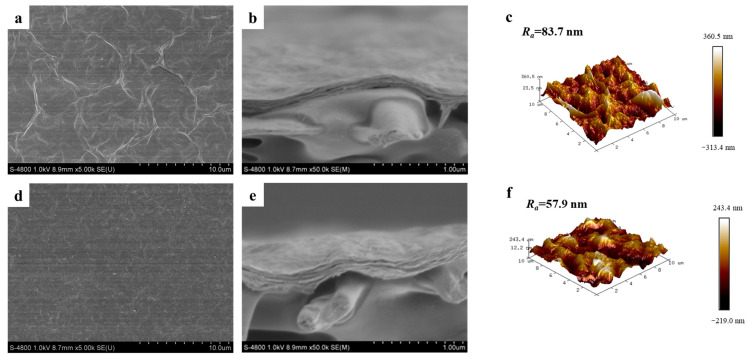
(**a**) Surface SEM image of MP0 membrane; (**b**) Cross-sectional SEM image of MP0 membrane; (**c**) AFM image of MP0 membrane; (**d**) Surface SEM image of MP50 membrane; (**e**) Cross-sectional SEM image of MP50 membrane; (**f**) AFM image of MP50 membrane.

The morphologies of the prepared Ti_3_C_2_-based membranes were observed by SEM. Figure 4a illustrates a characteristic corrugated structure observed in the pristine Ti_3_C_2_ (MP0) membrane. This unique morphology arises from the stacking and subsequent shrinkage of the nanosheets that occurs throughout the drying process [41]. Notably, the surface of the PEI-modified Ti_3_C_2_ (MP50) membrane does not exhibit any discernible corrugated morphology (Figure 4d), which is also reflected in AFM images. The average surface roughness (Ra) of the MP0 membrane is higher than that of the MP50 membrane, as depicted in Figure 4c,f. PEI molecules play important roles in the deposition process of Ti_3_C_2_, facilitating the uniform deposition of nanosheets and thereby contributing to the formation of a smooth surface. Lower roughness leads to a diminished effective filtration area and improved antifouling property. Meanwhile, the thickness of the Ti_3_C_2_ membrane increases a little after PEI modification seen from the cross-sectional images (Figure 4b,e), which may affect the separation performance.

**Figure 5:**
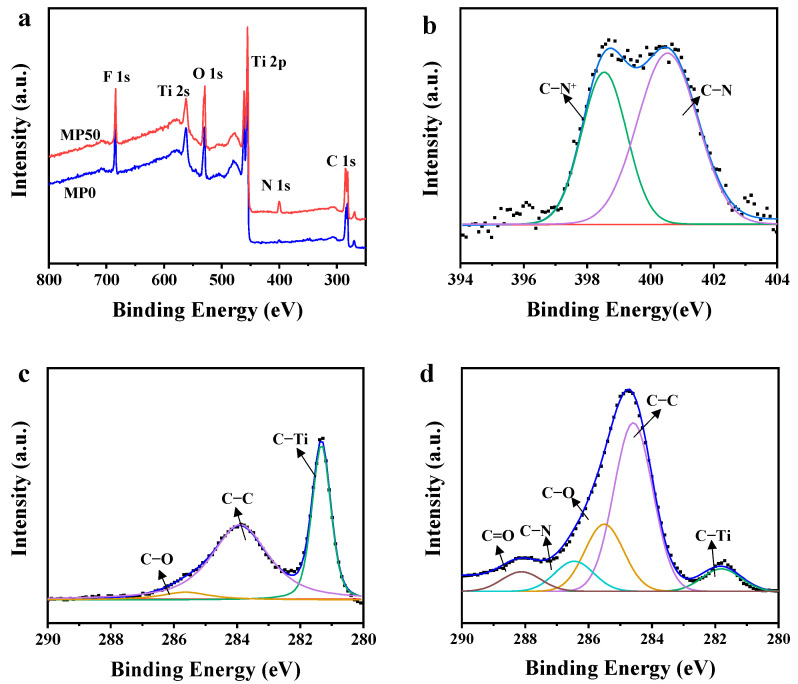
(**a**) XPS survey spectra of MP0 and MP50 membranes; (**b**) High-resolution XPS spectrum for the N 1s of the MP50 membrane; (**c**) High-resolution XPS spectrum for the C 1s of the MP0 membrane; (**d**) High-resolution XPS spectrum for the C 1s of the MP50 membrane.

The chemical composition of the membrane surface was investigated by XPS. As depicted in Figure 5a, the XPS survey spectrum for the MP0 and MP50 membranes reveals notable differences. Compared with the MP0 membrane, the MP50 membrane displays the emergence of a new peak of nitrogen. As shown in Figure 5b, the high-resolution XPS spectrum of the N 1s for the MP50 membrane is characterized by two distinct peaks at 398.6 eV and 400.9 eV, attributed to –NH_2_ and −NH_3_^+^ chemical bonds from PEI, respectively. Figure 5c illustrates the high-resolution XPS spectrum for the C 1s of the MP0 membrane, which presents three peaks at energies of 281.6, 284.1, and 285.8 eV, corresponding to C−Ti, C−C, and C−O bonds, respectively. For the MP50 membrane, as shown in Figure 5d, the high-resolution spectrum of C 1s has been further resolved into five peaks at 281.6 eV, 284.2 eV, 285.8 eV, 286.4 eV, and 288.2 eV, which correspond to C−Ti, C−C, C−O, C−N, and C=O bonds [42], respectively. In addition, after PEI modification, a decrease in the intensity of the C−Ti peak is observed, while the intensity of the C−C peaks shows an increase. These results confirm the successful incorporation of PEI into the Ti_3_C_2_ membrane.

**Figure 6:**
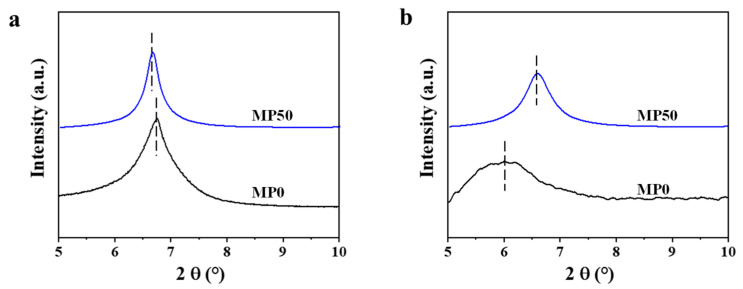
(**a**) The XRD pattern of the MP0 and MP50 membranes in the dry state; (**b**) The XRD pattern of the MP0 and MP50 membranes in the wet state.

The distance between neighboring nanosheets (d-spacing) can be determined using X-ray diffraction (XRD). As depicted in Figure 6a, the MP0 membrane displays a peak (002) at 2θ = 6.77°, indicating a d-spacing of approximately 1.31 nm. In contrast, the (002) peak for the MP50 membrane is observed at a slightly lower angle (2θ = 6.66°), suggesting that the incorporation of PEI into the Ti_3_C_2_ membrane expands the d-spacing to about 1.33 nm. These wider channels can improve the permeability of the membrane. As shown in Figure 6b, the MP0 membrane exhibits a notable increase in d-spacing when it is wetted, changing from approximately 1.31 nm to 1.47 nm. When placed in an aqueous solution, the water molecules that are absorbed in the nanochannels can cause unwanted swelling, which increases the interlayer spacing [29]. However, the d-spacing of the wetted MP50 membrane undergoes a slight increase, altering from approximately 1.33 nm to 1.35 nm. This is because PEI molecules can strengthen the interaction between neighboring nanosheets via bridging, which mitigates the swelling of the membrane.

### 3.3. Performance of Ti_3_C_2_-Based Membranes

**Figure 7:**
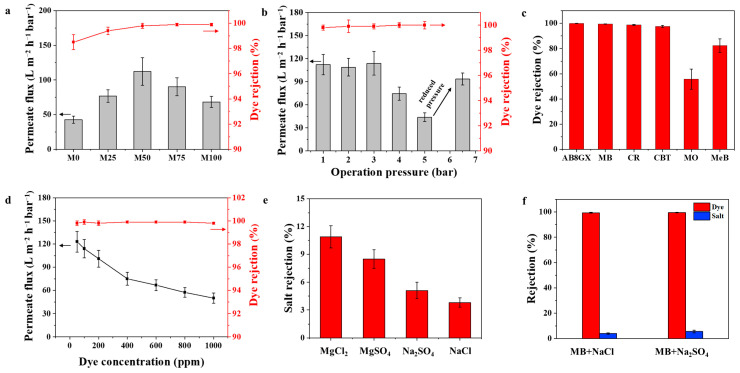
(**a**) The flux and the rejection rate for the prepared membranes using a 100.0 ppm MB solution at 3.0 bar pressure; (**b**) The flux and rejection rate for the MP50 membrane at varying operational pressures using a 100.0 ppm MB solution; (**c**) The rejection rate of the MP50 membrane when utilizing a feed of 100.0 ppm solutions with various dyes at 3.0 bar pressure; (**d**) The flux and the rejection rate for the MP50 membrane with differing concentrations of MB dyes at 3.0 bar pressure; (**e**) The rejection rate of the MP50 membrane to various salts at 100.0 ppm at 3.0 bar pressure; (**f**) The rejection rate of the MP50 membrane in a mixture containing dye and salt at 3.0 bar pressure (100.0 ppm each of dye and salt).

Figure 7a demonstrates that the permeate fluxes of Ti_3_C_2_-based membranes with different amounts of PEI initially increase before subsequently declining. The improvement in permeate fluxes can be associated with the greater distance between the layers. Nevertheless, a high content of PEI (greater than 50%) would obstruct the nanochannel and enhance the membrane’s thickness, consequently elevating the resistance to mass transfer. In addition, all the PEI-modified membranes show a better MB rejection than the pristine Ti_3_C_2_ membrane does. The PEI modification increases the size of the nanochannels, while the interactions (hydrogen bond and electrostatic force) between the PEI and Ti_3_C_2_ nanosheets enhance its anti-swelling performance. The contrasting effects of PEI on the structure of the membrane prevent the Ti_3_C_2_ laminates from being compressed excessively or being too loosely arranged. This modulation successfully alters the spacing between layers to an ideal nanoscale range, enabling the infiltration of water molecules while simultaneously limiting the movement of dye molecules, thereby addressing the trade-off limitation. Of these, the MP50 membrane has been selected for additional separation experiments.

The performance of the MP50 membrane was evaluated under various operating pressures. As illustrated in Figure 7b, the permeate flux of the MB solution rises with an increase in pressure from 1 to 3 bar; however, once the pressure surpasses 3 bar, the flux begins to decline. This phenomenon occurs because the PEI molecules become compressed as the pressure increases, leading to a reduced d-spacing value and narrower nanofiltration channels. When the test pressure is reduced from 5 bar to 1 bar, the flux recovers to 93.5 L m^−2^ h^−1^ bar^−1^, while it remains lower than the flux observed at the initial test pressure of 1 bar. The elasticity of hydrated PEI enables the restoration of the compressed channel as the pressure decreases. However, the nanochannels formed by the wrinkles in Ti_3_C_2_ membranes collapse under high pressure [43], and this process is irreversible, which causes the reduced flux. Thus, this Ti_3_C_2_-based membrane is particularly suitable for low-pressure nanofiltration applications with high efficiency. Furthermore, at all tested operating pressures, the rejection rate for MB dye by the MP50 membrane remains consistently high, exceeding 99.0%, which indicates the membrane’s remarkable stability.

A total of six dye types, differing in their molecular weights and charge characteristics, were employed to assess the separation capabilities of the MP50 membrane. Figure 7c illustrates that the MP50 membrane demonstrates a rejection rate exceeding 95% for dyes that possess different charges but have larger molecular weights (greater than 461.4 g mol^−1^). Specifically, the rejection rates are 99.9% for AB8GX (positively charged, M_w_ = 1298.9), 98.5% for MB (negatively charged, M_w_ = 799.8), 97.8% for CR (negatively charged, M_w_ = 696.7), and 95.6% for CBT (negatively charged, M_w_ = 461.4). In contrast, for dyes that possess low molecular weights, the rejection rates drop to below 90%. These findings indicate that the sieving effect significantly contributes to molecular filtration. In addition, the rejections of the MP50 membrane toward MO (negatively charged, M_w_ = 327.0) and MeB (positively charged, M_w_ = 319.1) are 55.8% and 82.3%, respectively, which is attributed to the electrostatic repulsion effects. In conclusion, both the effects of size sieving and Donnan repulsion work together to significantly influence the effective separation of dyes. In addition, textile wastewater generally has a significant concentration of dyes; thus, the impact of dye concentration on the efficiency of removal and the permeability of the membrane was examined. As illustrated in Figure 7d, the MP50 membrane exhibits a consistently high MB rejection rate (>99.0%), when the MB concentration changes between 50 and 1000 ppm. However, there is a notable reduction in permeate flux as the concentration of MB rises, resulting from enhanced concentration polarization and the accumulation of foulants on the membrane’s surface [44]. As shown in Figure 7e, the MP50 membrane exhibits a low level of salt rejection (<10%). An experiment aimed at separating dyes and salts, simulating real textile wastewater, was conducted. Figure 7f illustrates that the MP50 membrane exhibits a high rate of dye rejection while displaying a low rate of salt rejection in the mixed solutions. The MP50 membrane is capable of efficiently separating dye from salt, mainly due to its adjustable d-spacing, which aligns with the molecular sizes of both the dye and the salt. Significantly, as illustrated in Table 2, the MP50 membrane demonstrates superior dye rejection when contrasted with the most recent Ti_3_C_2_-based membranes reported in the literature, while also sustaining a high flux, highlighting its outstanding capacity for dye/salt separation.

### 3.4. Long-Term Stability of Membranes

**Figure 8:**
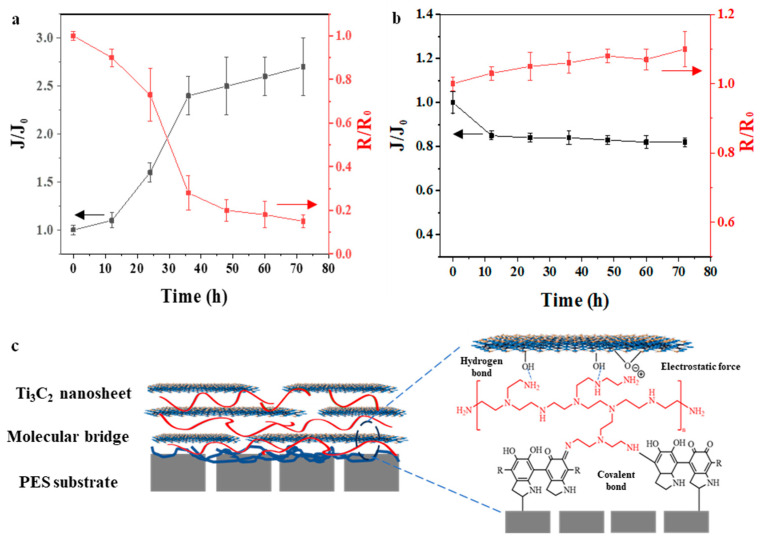
(**a**) Variations in permeate flux and rejection of Ti_3_C_2_/PES membrane over time (J_0_ = 52.8 ± 6.2 L m^−2^ h^−1^ bar^−1^, R_0_ = 98.5%, operation pressure = 3.0 bar, 100.0 ppm of MB solution as feed); (**b**) Variations in permeate flux and rejection rates of the MP50 membrane over time (J_0_ = 114.0 ± 15.5 L m^−2^ h^−1^ bar^−1^, R_0_ = 99.6%, operation pressure = 3.0 bar, 100.0 ppm of MB solution as feed); (**c**) Stabilizing a Ti_3_C_2_ membrane through molecular bridges.

The long-term stability of the membrane has been illustrated through the changes in membrane separation performance over a period of operation. As indicated in Figure 8a, a characteristic swelling-induced permeation pattern is evident in the Ti_3_C_2_/PES membrane; this pattern shows a gradual reduction in rejection rates alongside an increase in permeance throughout the treatment period. Notably, after 72 h treatment, the dye rejection of the Ti_3_C_2_/PES membrane decreases to only 20%. This reduction occurs because the Ti_3_C_2_ laminate tends to separate easily from the PES substrate under the crossflow mode due to insufficient adhesion. In contrast, the MP50% membrane shows a decline in permeance during 72 h treatment, which is mainly caused by the membrane fouling (Figure 8b). Meanwhile, the dye adsorbed on the membrane surface further enhanced the rejection. The long-term filtration tests reveal the exceptional stability of the MP50 membrane. The presence of PEI stabilizes the nanosheets through both hydrogen bonding and electrostatic interactions, thereby improving the membrane’s anti-swelling capacity. Moreover, the PDA coating provides ample physical interaction between the PEI−Ti_3_C_2_ laminate and the substrate. Furthermore, PDA promotes chemical bonding with the amine groups of PEI via a Schiff base reaction, thus reinforcing the bond between the substrate and the nanosheets (Figure 8c). Consequently, the bridging strategy is a crucial process for imparting high stability to the membrane.

## 4. Conclusions

In summary, we have shown a simple and effective approach to stabilizing Ti_3_C_2_ membranes through the formation and adjustment of molecular bridges. The intercalation of PEI enlarges the nanochannels of Ti_3_C_2_ laminate and inhibits the swelling of the nanosheets; polydopamine coating on the substrate surface enhances the force between the substrate and nanosheets, endowing the membrane with excellent combination characteristics of high nanofiltration performance and stability. The water permeance of the optimized MP50 membrane is 1.6 times improved, which is attributed to the expanded nanochannels. In addition, the adjustable nanochannels endow a membrane that demonstrates significant dye rejection (>95%) while maintaining low salt rejection (<10%). Moreover, the MP50 membrane shows exceptional long-term stability during water separation processes. This work presents a reliable strategy for stabilizing MXene membranes, opening new avenues for the design of promising high-performance 2D membranes in energy and environmental fields.

## Figures and Tables

**Table 1:** Composition of prepared membranes.

Membrane	Ti_3_C_2_ Loading (mg)	PEI Loading (mg)	PEI/Ti_3_C_2_ Ratio (%)
MP0	1.0	0	0
MP25	1.0	0.25	25
MP50	1.0	0.50	50
MP75	1.0	0.75	75
MP100	1.0	1.0	100

**Table 2:** Comparative filtration performance of the MXene-based membranes.

Membrane	Water Permeance (Lm^−2^ h^−1^ bar^−1^)	Dye Rejection (%)	Reference
MP50	112.3	AB8GX:99.9% MB: 99.5% CR: 97.8%	This work
MCE/MXene	44.97	MeB: 100.0%	[45]
MXene/ZIF-L	287.3	CR: 99.2%	[46]
PAA-MXene	516.3	AB8GX: 99.5%	[33]
MXene/PDA/PEI	38.4	CR: 99.7%	[47]
21%-Ag@MXene	420	RB: 79.9%	[48]
MXene-ANF	195.3	AB: 99.1%	[49]

## Data Availability

The original contributions presented in this study are included in the article. Further inquiries can be directed to the corresponding author.
